# OpenTrack simulation model files and output dataset for a copenhagen suburban railway

**DOI:** 10.1016/j.dib.2019.103952

**Published:** 2019-04-28

**Authors:** Steven Harrod, Fabrizio Cerreto, Otto Anker Nielsen

**Affiliations:** a Department of Management Engineering, Technical University of Denmark, 2800 Kgs. Lyngby, Denmark; b Banedanmark, Carsten Niebuhrs Gade 49, 1577 Copenhagen, Denmark

## Abstract

This data article offers two reference simulation models for the evaluation of performance and delays of a suburban railway line. Further, a sample output dataset is provided for validation of the model, and the sample output dataset could be used independently if the reader desires. The simulation is programmed in the OpenTrack railway simulation software Nash et al., 2004 ([2]). The models are provided as a reference and tool for other researchers to validate and extend their studies in railway operations.

These models were originally created for the research article “A Closed Form Railway Line Delay Propagation Model” Harrod et al., 2019 [1]. That article proposes an analytical estimation of railway delay, and these models were used to validate the findings.

**Table:** Specifications table

Subject area	*Transport, Railways, Simulation*
More specific subject area	*Railway Timetabling*
Type of data	*OpenTrack simulation models and output files*
How data was acquired	*The models were created by the authors. The performance of the trainsets was calibrated to match actual Copenhagen suburban trainsets of the SA/SE class in consultation with operator DSB. The dimensions and signal locations of the track closely follows the actual public information of the suburban railway from Hellerup to Hillerød, Denmark, in the year 2015.*
Data format	*The model files are a proprietary OpenTrack format. Selected output results are presented in an Excel spreadsheet.*
Experimental factors	*The experimental factors in running the models are selection of distribution and magnitude of primary delays and changes to the timetables of the trains.*
Experimental features	*The simulation model correctly calculates the speed and position of trains throughout their journey, and enforces safety rules and observation of signals that separate the trains. The timetable may be freely modified, and the results of a simulation viewed graphically and numerically. The primary output file of interest would be the text file “OT_Timetable.txt”, which stores a log of the train times at timing points.*
Data source location	*Banedanmark, Carsten Niebuhrs Gade 49, 1577 Copenhagen, Denmark;* *DSB, Telegade 2, 2630 Taastrup, Denmark*
Data accessibility	*The railway modeled has been revised since the models were created, and no longer has the same physical characteristics, and the track maps are thus no longer available. The rolling stock is still in service, and its performance data is available from DSB and other regulatory sources.*
Related research article	*Harrod, Steven, Fabrizio Cerreto, and Otto Anker Nielsen. 2019. “A Closed Form Railway Line Delay Propagation Model.” Transportation Research Part C 102. Elsevier Ltd: 189–209.*https://doi.org/10.1016/j.trc.2019.02.022*.*

**Table:** 

**Value of the data** • Model of a typical suburban railway from 2014, with technology and environmental characteristics comparable to many other railways. • Accurate representation of actual track segments and signal installation (a microscopic model). • The model simulates train departures to a timetable, inserts stochastic delays, and then reports the actual progress of the trains as they follow the signal rules and constraints of the railway line. • The models are suitable both for evaluation of performance (punctuality, delays), and for the evaluation of proposed timetables (robustness of timetables).

## 1 Data

This article presents OpenTrack railway simulation models that were originally composed to validate the results of an analytical delay estimation function in [1]. The primary output of interest is produced by the model in file “OT_Timetable.txt”, but the model also produces graphical figures showing the movement and track resource occupation of the trains. Sample output data is documented in the spreadsheet “Harrod 2019 DIB sample output”, and examples of graphical analysis are provided.

Two separate railway simulation models are included, both programmed in the commercial OpenTrack railway simulation software [2]. These models were developed in OpenTrack version 1.9, but they should be compatible with later versions. Both of these models are microscopic simulation models, where track is modeled to the meter and trains are controlled according to correct motion dynamics and signal rules. First, a “Generic” model of theoretical, homogeneous design, and second the “Hillerødbane”, a realistic, close to exact, model of the Hillerød suburban railway in Copenhagen. The Hillerødbane represents the railway as it was in the year 2015. The railway has since been reconstructed, and no longer has the same physical properties, however the same technology and operating patterns continue on many suburban railways worldwide.

The supplied model files are organized in folders “Test Generic for TRC” and “Test Hillerød for TRC”, and then the spreadsheet is provided separately. The folders are organized according to the recommendations in the OpenTrack documentation.

## 2 Experimental design, materials and methods

The two simulation models represent a single direction of traffic on a railway with discrete track blocks and fixed signals. The signals are configured with very long (1600 m) sight distances to emulate onboard cab signals with advance signal information. This type of technology and configuration is very common on many railways worldwide with technology dating from the 1960s–1990s. It is relatively easy to modify the models to represent newer technology, but this has not been done. Both simulation models accurately recreate the acceleration and braking of the SA/SE class of electrified suburban trainsets in Copenhagen. All simulation runs were configured with an initial delay at the first station drawn from a uniform distribution of [0, 600] seconds, but this is easily changed to other magnitudes and also to exponential or piecewise distributions (settings found in the “train categories” dialog).

The “Generic” railway model consists of a series of 10 stations. Each station consists of a “home” signal, 500 m of track, a stop position representing the platform, 50 m of track, and an exit signal. Between stations, there are four track segments of 1000 m, each with an intermediate block signal. The timetabled trains in this model are identical, and make all station stops.

The “Hillerødbane” model has the same technical and structural characteristics as the Generic model, but the track lengths, signal positions, and station locations are all unique to match the actual published track diagram of the railway from Hellerup to Hillerød, Denmark, in 2015. This railway has since been reconstructed and the signals completely changed to CBTC, a new technology that does not have fixed signal locations. The timetabled trains in this model have two configurations. There is a local train making all stops from Hellerup to Holte, and an express train making limited stops between Hellerup and Holte, and then all stops onward to Hillerød. It is relatively easy to modify the timetable in the OpenTrack software and this is not a fixed characteristic of the model.

In the research article [1], 100 replications starting from delay scenario 1 were generated, but the user can easily specify their own desired replication count, and also run the model without any stochastic delays by selecting “none” for the delay scenario. The timetables in the sample model files should not be treated as checked and validated timetables from the related research article [1]. They are just the last used timetable from the last user of the model. Users attempting to replicate the publication should check and edit the timetable to match the specifications in the research article. Full “OT_Timetable.txt” output is provided in the spreadsheet file for validation.
